# Ragnarok: A flexible and RApid GeNe annotation (ROcKs) pipeline deployed through Nextflow

**DOI:** 10.1002/tpg2.70338

**Published:** 2026-11-14

**Authors:** Ryan D. Kuster, Zane C. Smith, Lauren Whitt, Margaret Staton, Ben N. Mansfeld, Christopher Gottschalk

**Affiliations:** ^1^ Department of Entomology and Plant Pathology University of Tennessee Knoxville Tennessee USA; ^2^ USDA‐ARS Appalachian Fruit Research Station Kearneysville West Virginia USA; ^3^ HudsonAlpha Institute for Biotechnology Huntsville Alabama USA; ^4^ Department of Biology Washington University in St. Louis St. Louis Missouri USA

## Abstract

High‐quality genome assemblies and pan‐genomes are increasingly achievable and accessible due to advances in third‐generation sequencing and assembly algorithms, but genome annotation remains a critical bottleneck. Existing gene annotation pipelines often require complex installations, multiple steps, and long runtimes and produce variable results, which impede quality and the downstream usage of the annotations. We developed RAGNAROK (RApid GeNe Annotation ROcKs), a modular annotation pipeline built on Nextflow and Apptainer that integrates ab initio prediction, transcriptome and protein evidence, repeat annotation, functional assignment, and quality assessment into a single reproducible workflow for plant genomes. Moreover, RAGNAROK utilizes graphical processing unit acceleration and parallelization of select processes to decrease the time required to produce high‐quality gene annotation. We benchmarked against BRAKER3 across seven diverse, reference‐quality plant genomes and demonstrated that when including untranslated region (UTR) features, RAGNAROK achieved higher sensitivity, precision, and F1 scores at the exon, transcript, and gene levels. Furthermore, we demonstrated RAGNAROK's improvement in re‐annotating a suite of five Rosaceae genomes that were previously annotated using MAKER. Overall, RAGNAROK produced more ideal mono:multi‐exonic gene ratios, improved benchmarking of universal single‐copy orthologs completeness scores, and reduced missing gene content compared to other pipelines. Additionally, RAGNAROK consistently outperformed BRAKER3 in overall runtime, scaling efficiently from small to gigabase‐scale genomes. RAGNAROK provides a flexible, rapid, scalable, and accurate solution for de novo (first attempt or without prior evidence) and re‐annotation of plant genomes. Its modular design and workflow scalability lay the foundation for future extensions to animal, fungal, and other eukaryotic genomes. RAGNAROK is available at https://github.com/ryandkuster/ragnarok.

## Plain Language Summary

Improvements in sequencing the complete genome of plants continue, but identifying which regions of these sequences actually harbor genes and accurately annotating underlying gene structure is still non‐trivial and time‐consuming. Genome annotation remains slow and cumbersome, requiring numerous tools and processing steps. The proposed software pipeline RAGNAROK aims to overcome the challenges with current annotation pipelines through accelerating gene identification, easier use, and improved accuracy. The tool combines several methods for identifying genes into one streamlined Nextflow command that runs all required steps automatically. When tested against existing annotation software on seven different plant genomes, RAGNAROK performed better at correctly identifying genes and their individual components. When used to re‐analyze five previously studied fruit tree genomes, RAGNAROK found more complete genes and had fewer missing gene components compared to the original analysis.

**Abbreviations**
ANOVA: analysis of variance
BUSCO: benchmarking universal single‐copy orthologs
CDS: coding DNA sequence
CPU: central processing unit
GFF: general feature format
GO: gene ontology
GPU: graphics processing unit
GTF: gene transfer format
HPCC: high‐performance computing cluster
LRR: leucine‐rich repeat
LTR: long terminal repeat
NLR: nucleotide‐binding domain leucine‐rich repeat
SNAP: Semi‐HMM‐based Nucleic Acid Parser
TE: transposable elements
UTR: untranslated region

## 1 INTRODUCTION

With recent advancements in third‐generation sequencing technologies and improved genome assembly software, the ability to assemble high‐quality eukaryotic genomes has become increasingly accessible and achievable (Li & Durbin, 2024). These advances have rapidly increased genomic resources for species with large, heterozygous, and repetitive genomes, including plants (Gladman et al., 2023; Li & Harkess, 2018). However, genome assembly is only the first stage of generating a high‐quality genomic resource. Annotation of genic and repeat‐rich regions is currently the most time‐intensive and computationally demanding step (Dominguez Del Angel et al., 2018; Jung et al., 2020; Kim & Kim, 2022). The requirements of annotation are amplified in the pan‐genomic context, where uniformly annotating several to tens of complex genomes becomes crucial. Despite recent advances, producing accurate, reproducible, and accessible genome annotations remains a challenge.

The process of genome annotation begins with identifying the intergenic space, which consists of low‐complexity sequences that harbor transposable elements (TE), simple sequence repeats, pseudogenes, and other non‐coding features. Many tools have been developed to annotate specific features within genomes, such as Generic Repeat Find, HelitronScanner, and LTR_FINDER (where LTR is long terminal repeat) (Shi & Liang, 2019; Xiong et al., 2014; Xu & Wang, 2007). Outputs of these repeat‐identifying tools are often used as inputs into other software, such as RepeatModeler2 and LTR_retriever (Flynn et al., 2020; Ou & Jiang, 2018), to refine predictions of new or more complex related features. Over time, all‐in‐one pipelines or online servers that rely on such software have become available, providing greater ease‐of‐use (Baril et al., 2024; Humann et al., 2019; Ou et al., 2019; Su et al., 2021; The Galaxy Community, 2024).

Another annotation challenge is the identification of nucleotide‐binding leucine‐rich repeat (LRR) genes (commonly referred to as *R* genes). *R* genes inherently contain repetitive sequences and are commonly found in repetitive clusters within repeat‐rich regions (Bayer et al., 2018; Ellis et al., 2000; Hammond‐Kosack & Jones, 1997; Young, 2000), which are challenging even for evidence‐based alignments (proteins and RNA) of state‐of‐the‐art annotation tools. These arrays can be further confounded by the presence of sequence‐similar pseudogenes, which can serve as regulatory elements (Balakirev & Ayala, 2003; Pink et al., 2011). To overcome these challenges, *R* gene‐specific annotation pipelines such as nucleotide‐binding domain leucine‐rich repeat proteins (NLR)‐parser, NLR‐Annotator, and FindPlantNLRs have been developed (S. H. Chen et al., 2023; Steuernagel et al., 2015). Typically, these pipelines are executed on an unmasked genomic sequence to identify *R* genes. However, these tools have not yet been integrated into mainstream gene annotation pipelines, despite their ability to identify novel *R* genes missing from traditional gene annotations (Larson et al., 2025; Liu et al., 2025).

While low‐complexity repetitive space remains challenging to annotate, high‐complexity genic space is even more onerous. Many tools have been developed to recognize sequence features of protein‐coding genes and predict gene annotations without evidence, such as GeneMark, Semi‐HMM‐based Nucleic Acid Parser (SNAP), Augustus, Helixer, and Tiberius (Brůna et al., 2020; Gabriel, Becker, et al., 2024; Holst et al., 2025; Korf, 2004; Stanke et al., 2004; Stiehler et al., 2021). Ab initio gene predictors can improve their accuracy and sensitivity for identifying features when iteratively trained (i.e., GeneMark, SNAP, and Augustus). However, this process is time‐consuming and requires substantial user interventions if not automated with a pipeline (e.g., MAKER). In comparison, some ab initio predictors, such as Helixer and Tiberius, rely on a preset inference dataset and require a single execution (Gabriel, Becker, et al., 2024; Holst et al., 2025; Stiehler et al., 2021). These tools are additionally graphics processing unit (GPU)‐accelerated, which offers substantial run‐time or wall‐time savings. In addition to ab initio predictions, annotation can also leverage evidence‐based inputs such as a de novo or reference‐guided transcriptome assembly. Transcriptome assemblers, such as Trinity and StringTie, can assemble transcriptional gene models from short‐read RNA‐seq data either de novo or from RNA‐seq reads aligned to the genome, respectively (Grabherr et al., 2011; Pertea et al., 2016). With the advent of third‐generation sequencers, long‐read RNA‐seq (e.g., PacBio Iso‐Seq) can also be employed in transcriptome assembly by programs such as StringTie and RNA‐bloom2 (Kovaka et al., 2019; Nip et al., 2023; Shumate et al., 2022).

Beyond transcripts, protein sequence‐based evidence annotation is also useful. Tools such as Exonerate's protein2genome and miniprot are capable of aligning protein sequences (Li, 2023; Slater & Birney, 2005). Ultimately, most published genome reports utilize a combination of ab initio predictions and evidence‐based annotations. This combination method for gene annotation has been integrated into all‐in‐one annotation pipelines such as MAKER (prediction‐only, but evidence‐based), BRAKER (prediction and evidence‐based), and Funannotate (prediction‐only) (Campbell et al., 2014; Gabriel, Brůna, et al., 2024; Palmer & Stajich, 2020).

Although aimed at simplifying the genome annotation process, these all‐in‐one annotation pipelines can be challenging to install, cumbersome to execute, complex to parameterize, slow to run, and difficult to reproduce. For example, de novo annotations using BRAKER, where RNA‐seq and protein data are available, require genome annotations generated by BRAKER1 (RNA‐seq evidence‐based) and BRAKER2 (protein evidence‐based). The BRAKER team subsequently released the transcript selection tool TSEBRA, which integrates functionality developed in BRAKER1 and BRAKER2, to increase accuracy through complementary transcript selection (Gabriel et al., 2021). This procedure has since been integrated into a singular pipeline: BRAKER3 (Gabriel, Brůna, et al., 2024). Furthermore, under‐ and over‐annotation can be observed when using these pipelines. For example, the MAKER and BRAKER pipelines are often challenged by large, highly repetitive plant genomes and a lack of available evidence‐based data (Vuruputoor et al., 2023). Vuruputoor et al. (2023) demonstrated these flaws by re‐annotating the repetitive and large *Liriodendron* genome using various combinations of RNA‐seq evidence in the BRAKER and BRAKER + TSEBRA pipelines. Vuruputoor et al. (2023) reported a total of 30,035–51,804 annotated genes, compared to the published annotation of 35,261, when using different RNA‐seq inputs and filtering methods (J. Chen et al., 2019; Vuruputoor et al., 2023), demonstrating the high variability in annotation.

To overcome the challenges associated with annotation, we developed a Nextflow pipeline that utilizes Apptainer to combine a suite of fast and accurate ab initio and evidence‐based gene annotators into a novel, all‐in‐one annotation pipeline (Kurtzer et al., 2017; Langer et al., 2025). This pipeline, named RApid GeNe Annotation (ROcKs) or RAGNAROK (https://github.com/ryandkuster/ragnarok), aims to be a rapid, flexible, accurate, and reproducible option for the de novo (re‐)annotation of plant genomes in the pangenome era. Moreover, RAGNAROK's implementation also allows for the optional incorporation of repeat annotation using EDTA before gene annotation. RAGNAROK also performs integrated gene model filtering, functional annotation, and quality assessment for convenient end‐user validation of genome annotation quality. We benchmarked RAGNAROK against other annotation pipelines (MAKER and BRAKER) on both model and non‐model genomes of variable ploidy to demonstrate its higher accuracy, precision, and overall annotation quality. By deploying our pipeline through the increasingly popular Nextflow workflow management system (Langer et al., 2025), we eliminate the need for users to install all dependencies, thereby creating a reproducible and scalable workflow.

## 2 MATERIALS AND METHODS

### 2.1 Pipeline development and implementation

RAGNAROK annotation is split into several parallel processes for annotation: (1) ab initio gene prediction, (2) evidence‐based (e.g., RNA‐seq) transcriptome assembly, (3) protein database mapping, and (4) optional liftover of previous annotations (Figure 1). The pipeline begins with collecting input sequences, which include an unmasked genome assembly FASTA, short‐ and/or long‐read RNA‐seq datasets, a protein dataset, and an optional coding DNA sequence (CDS) reference set for the target species or a close relative. During the initial development of RAGNAROK, we elected for the genome FASTA to undergo TE annotation using EDTA (v2.2.2) (Ou et al., 2019), which generates a masked genome FASTA that excludes TEs longer than 1 kb (hard‐masked) to use as input for all subsequent steps. This process is now optional but recommended. EDTA was executed using default parameters with the unmasked genome FASTA and CDS sequences for the target or related species as recommended by EDTA's developers (Ou et al., 2019).

**FIGURE 1:**
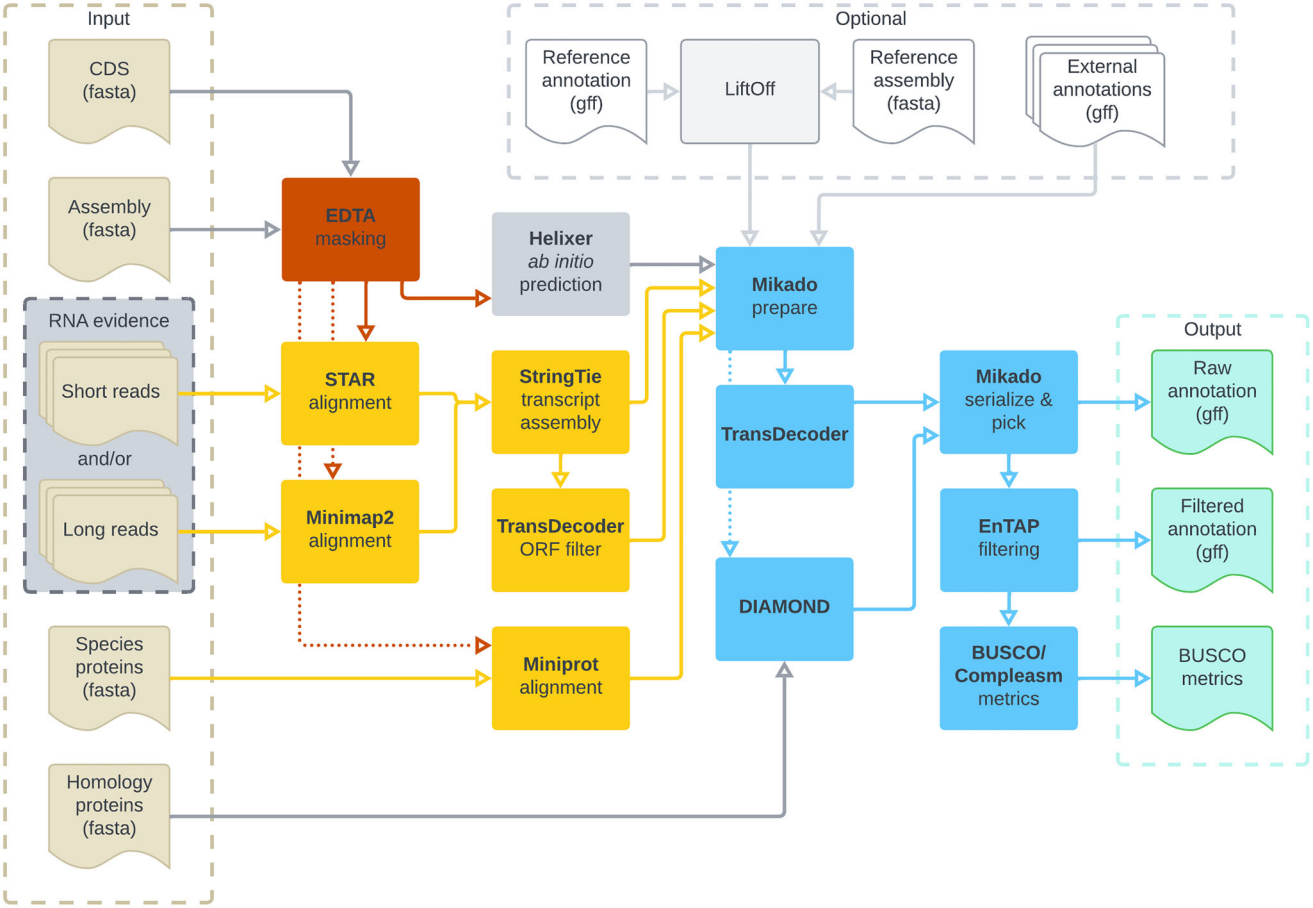
Flowchart of critical RAGNAROK pipeline stages. A reference genome assembly is optionally masked (red) before ab initio (Helixer) and evidence‐based alignment prediction steps (yellow). Post‐prediction steps (shown in blue) include weighted transcript selection, functional annotation, and benchmarking universal single‐copy orthologs (BUSCO) statistics.

For ab initio gene prediction, RAGNAROK leverages the GPU‐accelerated Helixer gene predictor (v0.3.4) (Holst et al., 2025; Stiehler et al., 2021), which maintains support for the central processing unit (CPU) implementation of Helixer at the expense of additional processing time. Here, the EDTA‐masked genome file and the Helixer scoring file are used as inputs into Helixer with default plant parameters (Holst et al., 2025; Ou et al., 2019; Stiehler et al., 2021).

For RNA‐seq‐based transcriptome assembly, we built RAGNAROK to concurrently utilize the StringTie pipeline (v3.0.0) with short‐ and/or long‐reads (Kovaka et al., 2019; Pertea et al., 2016; Shumate et al., 2022). With short‐read data, reads are first assessed for quality using FASTQC (v0.12.1) and MULTIQC (v0.24.1), then evaluated for adapters and trimmed by FASTP (v0.23.4) (Andrews, 2010; S. Chen et al., 2018; Ewels et al., 2016). A second assessment of the resulting trimmed reads is conducted with FASTQC and MULTIQC. The masked genome file is then indexed using the STAR aligner (v2.7.11a), and trimmed reads are mapped onto the genome using the following parameters ­‐outSAMtype BAM Unsorted ­‐outSAMstrandField intronMotif ­‐alignIntronMax 10000 (Dobin et al., 2013). With long‐read data, the masked genome is indexed using minimap2 (v2.28), and reads are aligned with the following parameters ‐ax splice:hq ‐uf enabled (Li, 2018). All aligned reads are then sorted using SAMtools (v1.20) and used as input into StringTie, with the hybrid mode enabled if long reads were used, to perform transcript assembly (Danecek et al., 2021; Kovaka et al., 2019; Pertea et al., 2015, 2016; Shumate et al., 2022). The resulting gene transfer format (GTF) annotation file is subsequently converted to GFF3 (where GFF is general feature format) format using gffread (v0.12.7) (Pertea & Pertea, 2020). We additionally use the StringTie GTF as input into TransDecoder (v5.7.1) (https://github.com/TransDecoder/). TransDecoder aims to extract the longest open‐reading frame transcripts from each locus in the StringTie output. Here, we first execute *gtf_genome_to_cdna_fasta.pl* using the StringTie GTF and masked genome file. We then executed *TransDecoder.LongOrfs* and *TransDecoder.Predict* using the output from *gtf_genome_to_cdna_fasta.pl*. The StringTie GTF is then used again as input into *gtf_to_alignment_gff3.pl*. The last step of the TransDecoder steps was *cdna_alignment_orf_to_genome_orf.pl* using the outputs from all three prior steps as suggested by TransDecoder's documentation (https://github.com/TransDecoder/). Additionally, RAGNAROK generates a second evidence‐based annotation from a reference protein dataset by indexing and aligning to the masked genome using miniprot (v0.13) with the −gff evoked (Li, 2023).

We also added an optional step for evidence‐based annotation via LiftOff (v1.6.3), which “lifts” over existing reference annotations from a similar species by selecting optimal exon alignments (Shumate & Salzberg, 2021). LiftOff is also particularly useful for transferring pre‐existing gene annotations from previous annotation versions. This step was executed using the default parameters and gene copy search to enable the retrieval of novel duplications, considering only alignments with at least 95% identity. Additionally, RAGNAROK flexibly supports the inclusion of user‐generated GFFs from other tools, for example, Cufflinks (Trapnell et al., 2012), Prothint (Brůna et al., 2020), or other annotation pipelines.

Following gene predictions and evidence‐based annotations, we used the Mikado (v2.3.5rc2) transcript selector to reduce redundancy and select the best representative transcript models (Venturini et al., 2018). Although written for polishing of reference annotations, Mikado can also provide a consensus annotation from de novo and ab initio gene predictions. The Mikado input file is a tab‐delimited list that includes the resulting GTF/GFFs from all previous annotation steps. By default, these inputs include the StringTie GTF, miniprot GFF, Helixer GFF, TransDecoder GFF, and, optionally, a LiftOff or user‐supplied GFF(s). The miniprot, StringTie, and optional LiftOff datasets were flagged as reference annotations. Our scoring for all inputs was scaled by our confidence in the annotation tools (StringTie = 10, miniprot = 10, Helixer = 1, TransDecoder = ‐0.5), but the user must include their own weights for the LiftOff GFF based on their confidence in those annotations. With the list file created, we then executed Mikado configure with the list file as the ­‐list input, the masked genome file as the ­‐reference input, and the Mikado prepared plants.yaml as the ­‐scoring input, the UniProt Swiss‐Prot protein sequences (The UniProt Consortium, 2025) as the ­‐homology input, and a modified Mikado plant.yaml.

By default, Mikado does not add penalties to any given line of evidence; however, we introduce a severe penalty for any models that contain microexons smaller than 3 bp in length, which are exceptionally rare (Islamov et al., 2025) and address known shortcomings of Helixer predictions (Brůna et al., 2026). The evidence weighting scheme for Mikado allows the user to adjust their confidence level for each line of evidence (i.e., Helixer, StringTie, miniprot, and TransDecoder) that produces a predicted gene model. After scoring and weighting, gene models scoring at or below 0 are removed from transcript selection, whereas gene models scoring above 0 are retained. Evidence weights are only applied after Mikado scores each gene model. After reweighting, overlapping gene models from the provided lines of evidence that score greater than 0 are compared, and the highest scoring model is selected by Mikado for the final annotation set.

At present, ab initio predictions are not a replacement for extrinsic evidence. Thus, we weight gene models with existing transcript or protein evidence with a much higher confidence than ab initio predictions by default. Our observations suggest that gene models typically score between 10 and 30, meaning evidence‐based predictions gain a substantial advantage over overlapping, ab initio predictions. In summary, our default evidence weighting procedure penalizes gene models that are biologically unlikely and rewards models that are based on extrinsic evidence, which we demonstrate performs well for a suite of plant species, including paleopolyploids (*Malus* spp.) with high levels of duplication. This approach supports our aim for RAGNAROK to prefer annotations based on extrinsic evidence while maintaining sensitivity for ab initio gene predictions in complex gene families, such as NLRs (Kourelis et al., 2021).

After transcript selection, we then executed Mikado prepare ­‐json‐conf using the modified Mikado yaml (plant_microexon_filter.yaml) (Venturini et al., 2018). Once completed, we then prepared a DIAMOND (v2.1.11) database using the UniProt Swiss‐Prot protein sequences (Buchfink et al., 2021). DIAMOND was then executed on the Mikado prepared transcript FASTA file with the following flags: blastx ‐k 5 ‐f 6 qseqid sseqid pident length mismatch gapopen qstart qend sstart send evalue bitscore ppos btop (Buchfink et al., 2021). Prior to continuing the Mikado pipeline, we used *TransDecoder.LongOrfs* on the Mikado prepared transcript FASTA file. Thereafter, Mikado serialise was executed using the Mikado prepared configuration.yaml as the ­‐json‐conf input, the Mikado prepared FASTA as ­‐transcripts input, the DIAMOND output at the ­‐xml input, and the *TransDecoder.LongOrfs* output was prepared on the Mikado transcript FASTA as the ­‐orfs input. Finally, the UniProt Swiss‐Prot proteins were used as the ­‐blast_targets input (Venturini et al., 2018). The final step was to execute the Mikado pick function with the following parameter inputs: the masked genome FASTA as the ­‐fasta input, Mikado configuration.yaml as the ­‐configuration input, and the ­‐subloci‐out and ­‐loci‐out flags enabled. The resulting loci‐out file was treated as a final, unfiltered gene annotation file (Venturini et al., 2018).

To further process and increase the evidence that a gene is annotated correctly, RAGNAROK carries out functional annotation. This functional annotation is conducted using EnTAP (v2.2.0) (Hart et al., 2020). Briefly, EnTAP conducts functional annotation by performing similarity searches of the protein to several databases using DIAMOND (Buchfink et al., 2021). We specifically use annotations from Swiss‐Prot and NCBI RefSeq Plant databases as references. Gene ontology (GO) terms are additionally assigned using EggNOG‐mapper (v2) and the EggNOG database (v6) (Cantalapiedra et al., 2021; Hernández‐Plaza et al., 2022). Following functional annotation, we created a custom script to further filter the annotations from Mikado to remove pseudogenes and mono‐exonic features that lack transcribed proteins. This custom filtering script relies on the AGAT (v1.4.1) subset functionality (Dainet, 2019).

To assess the quality of the resulting RAGNAROK annotation, we integrated several assessment tools. First, to determine the accuracy of annotating universal single‐copy orthologs, we utilized the benchmarking program benchmarking universal single‐copy orthologs (BUSCO) (5.8.2) (Manni et al., 2021; Simão et al., 2015) using the latest OrthoDB v12 databases (Tegenfeldt et al., 2025). We also implemented the complementary BUSCO evaluation tool, Compleasm, which is functionally similar to BUSCO with a reportedly higher sensitivity (Huang & Li, 2023).

### 2.2 Input data description for users

RAGNAROK has several required data inputs. The first is a genome FASTA that can be optionally TE‐masked using the ­‐perform_masking parameter. This will allow RAGNAROK to execute masking of TEs prior to gene annotation. The user may also supply a CDS FASTA from the target (or a related) species to increase EDTA's accuracy with the ­‐cds parameter. RAGNAROK also requires RNA‐seq data sourced from the target genome or a close relative, if applicable. The RNA‐seq data inputs are inclusive to sequencing platform with ­‐ill for Illumina paired‐end, ­‐pb for PacBio IsoSeq, and ­‐ont for Oxford Nanopore RNA/cDNA. One of these inputs is required but a user can input mixed data types (e.g., Illumina and IsoSeq). The last data input required is a protein FASTA with the ­‐protein parameter. This dataset may be sourced from the target species, a related species, or a representative dataset, such as Swissprot's Viridiplantae. In addition, the Mikado functions within RAGNAROK require three files: (1) protein homology files, (2) a scoring file, and (3) a design file. Examples and recommendations can be found in Mikado's documentation and on RAGNAROK's GitHub.

### 2.3 Benchmarking

To evaluate the performance of RAGNAROK for de novo annotation, we compared against BRAKER3 (v3.0.8) on seven plant genomes with reference gene annotations that have been either heavily curated or have undergone extensive annotation parameter testing (Gabriel, Brůna, et al., 2024). These genomes include *Arabidopsis thaliana* TAIR10, *Prunus persica* ‘Lovell 2D’ v2, *Malus domestica* GDDH13, *Zea mays* B73, *Liriodendron chinense*, *Solanum tuberosum* 686 v6, and *Triticum aestivum* ‘Chinese Spring’ v2.1 (J. Chen et al., 2019; Cheng et al., 2017; Daccord et al., 2017; Hufford et al., 2021; Lamesch et al., 2012; Pham et al., 2020; Verde et al., 2013, 2017; Wu et al., 2023; Zhu et al., 2021). We utilized the same TE annotation and masked genome assembly for each species to directly compare against BRAKER3. This was accomplished by using the EDTA mod.MAKER.masked (FASTA) output was generated within RAGNAROK as the masked genome input for BRAKER3, thus ensuring both pipelines received the same masked sequences. References accessed from Phytozome were masked in the same manner (TE > 1 kb) using pre‐existing TE annotations. For evidence datasets (e.g., RNA‐seq and protein databases), we evaluated two approaches: (1) the “best case” scenario that used identical reference protein databases and RNA‐seq data and (2) identical RNA‐seq data and an expansive protein database (OrthoDB Viridiplantae or Uniprot Viridiplantae) to simulate complete de novo annotation (Ahmad et al., 2025; Tegenfeldt et al., 2025). Unlike BRAKER3, which recommends OrthoDB as a protein database due to its use of homology‐based seeding, RAGNAROK performs exhaustive alignment against the full database, rendering OrthoDB computationally impractical as an input. As a result, a smaller representative protein database from Uniprot Viridiplantae was used as input into RAGNAROK. All corresponding commands for RAGNAROK and BRAKER3 for these seven genomes can be found in Supporting Information 1. Precision and sensitivity statistics of the two resulting de novo annotations per species were obtained using Mikado's stats function and BUSCO using OrthoDB v12 (Manni et al., 2021; Simão et al., 2015; Tegenfeldt et al., 2025; Venturini et al., 2018).

Comparisons between the annotations and the reference annotation of each species were obtained using Mikado compare feature (Venturini et al., 2018). Metrics from the resulting stats file were reported at the base level, exon level (lenient), transcript level (≥80% base F1), and gene level (≥80% base F1). Genomes, reference annotations, evidence datasets, and versions for each of the seven subject species can be found in Table S1 (J. Chen et al., 2019; Daccord et al., 2017; Hufford et al., 2021; Lamesch et al., 2012; Pham et al., 2020; Verde et al., 2013, 2017; Wu et al., 2023; Zhu et al., 2021). We additionally used the Mikado compare *tmap* output file to determine the novel loci and underlying transcripts (identified as n_f1 = 0). These novel transcripts were then analyzed for protein coding potential using PSAURON (Sommer et al., 2025). We also investigate each novel transcript for RNA‐sequencing coverage. Here, the annotation files for the Viridiplantae BRAKER3 and RAGNAROK runs were used as input into StringTie with the ‐G parameter and the ‐e parameter enabled to estimate expression per transcript using RNA‐seq alignments generated by STAR. The resulting GTF file was then filtered for the novel transcripts to obtain read coverages. We then calculated the percent of novel transcripts that PSAURON predicted to be non‐protein‐coding to the total number of novel transcripts. We took a similar approach in determining the percent of novel transcripts that lacked expression support (read coverage = 0).

We also compared the ability of RAGNAROK and BRAKER3 to annotate *R‐*genes. We compared the total *R‐*genes annotated by each pipeline from the Viridiplantae BRAKER3 and RAGNAROK annotation runs with the reference annotation for each of the seven species using Resistify (v.2.1.0) (Smith et al., 2025). Here, the extracted protein FASTAs were used as inputs into the resistify nlr command.

To ensure fair run‐time comparisons between RAGNAROK and BRAKER3, all seven reference‐quality genomes were annotated on identical computing hardware for each pairwise benchmark (either Twin Intel Xeon Gold 6252 @ 2.10 GHz, 96 threads, 192 GiB RAM, or AMD EPYC 9734 112‐Core Processor, 224 threads, 1007 GiB RAM). For RAGNAROK, the Nextflow script was executed using the local profile and eight (Supporting Information 1). For BRAKER3, all datasets were executed with the threads parameter set to 96 (Supporting Information 1).

We also evaluated RAGNAROK for re‐annotation of several non‐model, paleopolyploid species that were previously annotated using the MAKER (v2.31.10) pipeline (Campbell et al., 2014). These genomes included *Malus angustifolia, Malus fusca, P. persica* ‘Lovell 2D’ v3 and ‘Lovell 5D,’ and *Pyrus communis* ‘d'Anjou’ (Gottschalk et al., 2026; Mansfeld, Ou, et al., 2023; Mansfeld, Yocca, et al., 2023; Yocca et al., 2024). Similar to the de novo annotation, the same masked genome file and evidence datasets were used as input. Statistics for the RAGNAROK and MAKER annotations were obtained using Mikado stats function and BUSCO using OrthoDB v12 (Manni et al., 2021; Simão et al., 2015; Tegenfeldt et al., 2025; Venturini et al., 2018). Genomes, reference annotations, evidence datasets, and versions for each of the five subject species can also be found in Table S1. Statistical analysis was conducted using R (v.4.5.1) (R Core Team, 2024). For multiple comparisons, we conducted Kruskal–Wallis Rank Sum tests and paired Wilcoxon tests to determine significance, where appropriate. For the Wilcoxon tests, we additionally adjusted the *p‐*values using the Holm–Šídák adjustment. Figure images were generated using ggplot2 (Wickham, 2016).

## 3 RESULTS

### 3.1 Comparing de novo annotation of RAGNAROK versus BRAKER3

To evaluate the performance of RAGNAROK, we compared it to BRAKER3, the most widely used eukaryotic annotation pipeline (Gabriel, Brůna, et al., 2024). Here, we selected seven reference‐quality or thoroughly annotated plant genomes: *A. thaliana, P. persica, M. domestica, Z. mays, L. chinense, S. tuberosum*, and *T. aestivum* to serve as independent comparisons across diverse genome architectures. Each genome was treated as being de novo annotated using publicly available data that was either presented in the original publication of the genome (e.g., RNA‐seq) or in previous evaluations of BRAKER3 (Table S1). BRAKER3 also recommends utilizing a non‐species‐specific protein database (e.g., OrthoDB Viridiplantae) for de novo annotation. As a result, we approached protein inputs in two different ways: (1) best case scenario where a reference protein database for the species is available and (2) a true de novo approach using a non‐species‐specific database. To compare the quality of the annotations, the separate annotations generated by RAGNAROK and BRAKER3 were compared to the reference annotations for each species annotated (Table S1).

Overall, we observed higher sensitivity with RAGNAROK compared to BRAKER3 at the base, exon, transcript, and gene level when compared to the reference annotation of the genome (Figure 2; Table S2). BRAKER3 was more precise at the base and exon level but was less precise at the transcript and gene level. Significantly higher sensitivity was observed for RAGNAROK at the base, exon, transcript, and gene levels regardless of the protein database used. This trend was consistent for precision at the gene and transcript level regardless of the protein database. As a result, the F1 values (the harmonic mean of sensitivity and precision) were also found to be significantly higher for RAGNAROK annotations at the base, transcript, and gene levels regardless of the protein database used (Figure 3; Table S2).

**FIGURE 2:**
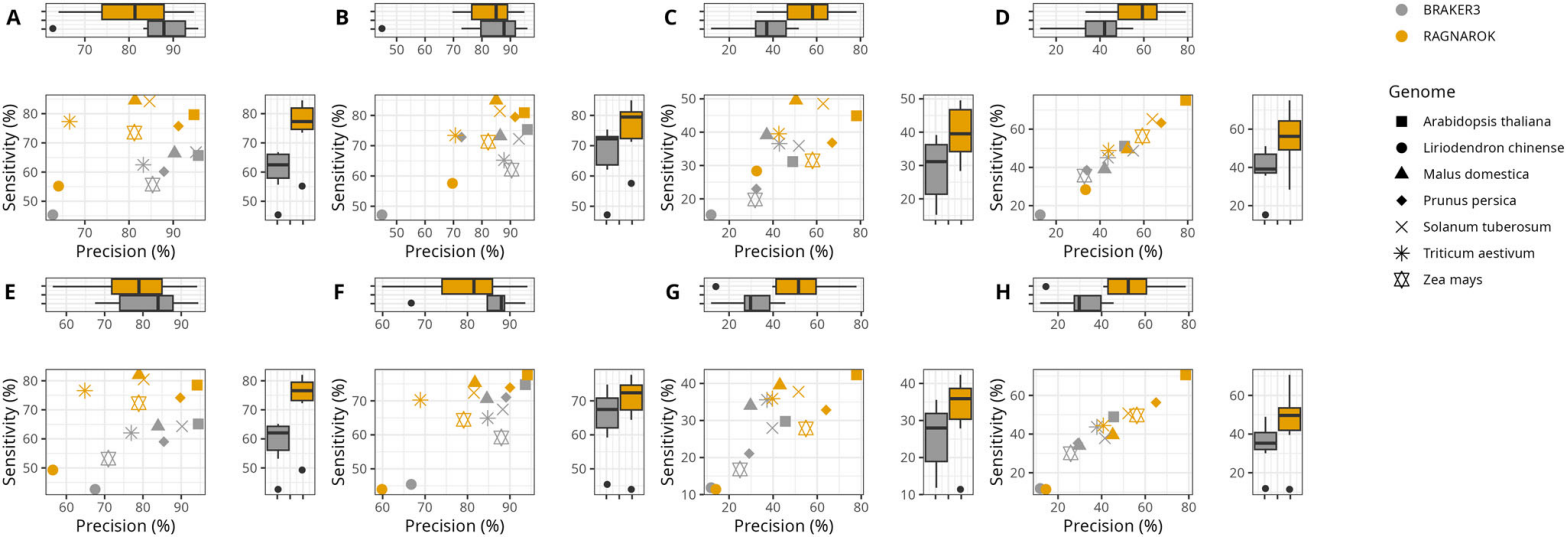
Precision and sensitivity at different levels of annotation from BRAKER3 and RAGNAROK compared to reference annotation for seven genomes using the reference protein database (A–D) and non‐species‐specific protein database (E–H). (A) Base level, (B) Exon level, (C) Transcript level, (D) Gene level, (E) Base level, (F) Exon level, (G) Transcript level, and (H) Gene level.

**FIGURE 3:**
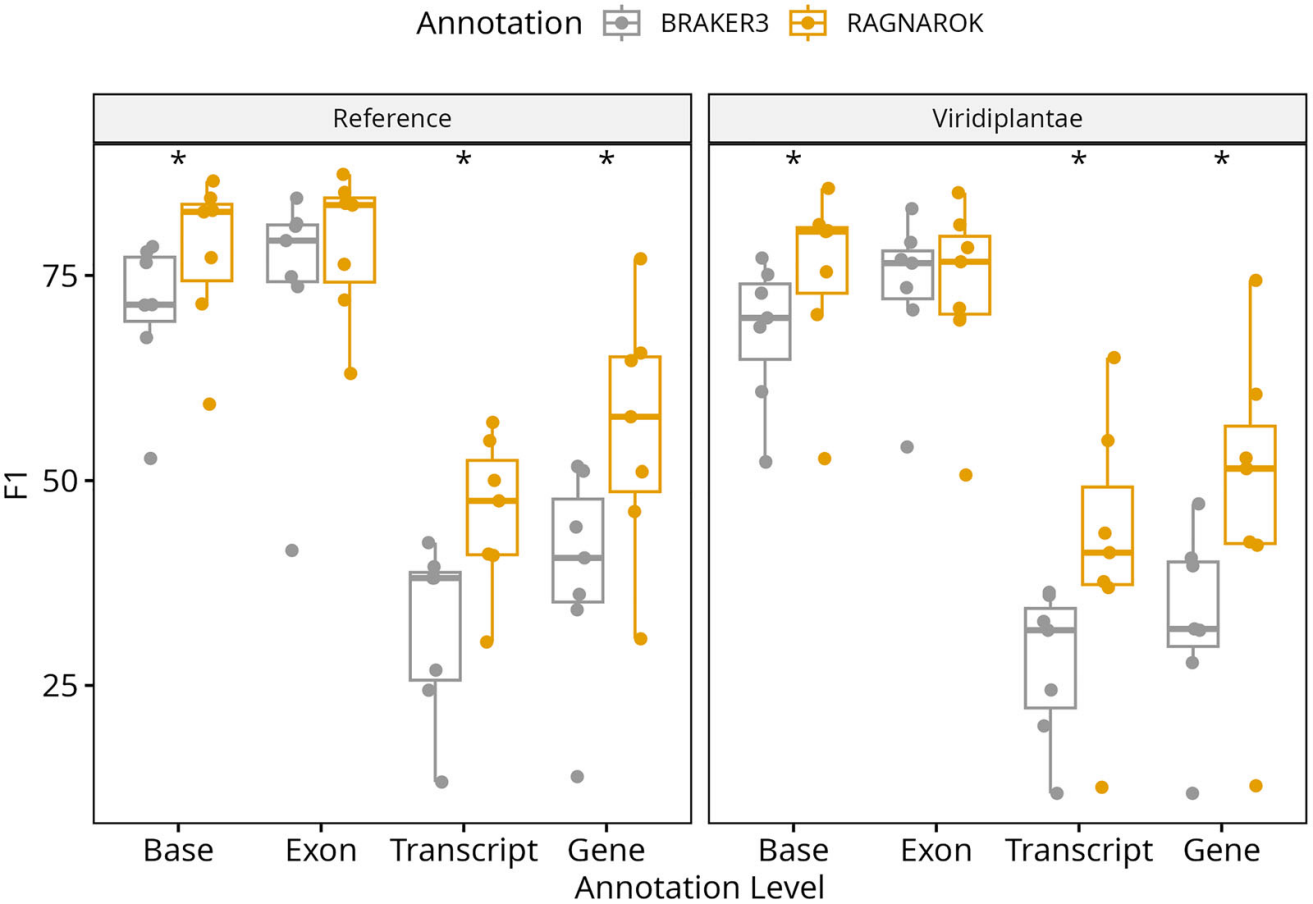
F1 scores between BRAKER3 and RAGNAROK when compared to the reference annotation for seven genomes using two different protein datasets. Asterisks denote significant differences between the F1 score distributions using a Wilcoxon test and Holm–Šídák adjustment, *p ≤ 0.05.

We also compared the resulting RAGNAROK, BRAKER3, and reference annotations for each of the seven species and two different input protein evidence datasets for the total number of all genes, only mono‐exonic genes, and the ratio of mono:multi‐exonic gene annotations (Table 1; Figure 4). Here, neither RAGNAROK nor BRAKER3 produced significantly different total gene annotations compared to the reference annotations. This result held true regardless of the input protein datasets used. When comparing the total mono‐exonic gene annotations, the Kruskal–Wallis test exhibited *p‐*values of 0.046 and 0.032 for the reference protein and Viridiplantae protein dataset inputs. In mono‐exonic gene annotations, RAGNAROK generally exhibited similar numbers to the reference annotation but was not significantly different. In contrast, BRAKER3 was significantly higher compared to RAGNAROK and the reference annotations, regardless of the protein dataset input, with an adjusted *p‐*value = 0.047. In association, the ratio of mono:multi‐exonic annotated genes was also significantly different with Kruskal–Wallis *p‐*value = 0.0011 and 0.0012 for the reference Viridiplantae protein dataset inputs, respectively. Again, BRAKER3 was significantly higher than RAGNAROK and the reference annotation (adjusted *p‐*value* = *0.047). Furthermore, RAGNAROK consistently had fewer missed exons compared to BRAKER3, except in one case of *Liriodendron* when using the Viridiplantae databases for protein input. When it came to missed genes, the results were more inconsistent. RAGNAROK consistently missed fewer genes when using the reference protein datasets as input. With the Viridiplantae databases, BRAKER3 exhibited lower missed genes for the *Arabidopsis, Prunus*, and *Liriodendron* genomes, whereas RAGNAROK exhibited fewer missed genes in the other four genomes. While the differences were not significant at the exon or gene level (Kruskal–Wallis *p‐*value = 0.15), it is notable that RAGNAROK demonstrated stronger performance on species with larger genomes and variable ploidy levels (Triticum and Solanum are polyploids; Malus is a paleopolyploid) (Pham et al., 2020; Velasco et al., 2010; Zhu et al., 2021). Polyploid and highly repetitive genome annotation merits further investigation as a distinct use case.

**TABLE 1:** Annotation statistics between RAGNAROK, BRAKER3, and reference annotation on seven reference quality genomes.

Annotation	Protein input	Number of genes	Mono‐ exonic genes	Mono: multi	Missed exons	Matched intron chains	Missed genes	Novel genes	*R* genes	BUSCO Embryophyta ODB12
***Arabidopsis thaliana***										
Araport11	–	27,655	5382	0.242	–	–	–	–	167	C:98.9 [S:48.7, D:50.2], F:0.4, M:0.6
BRAKER3	ref	27,596	7959	0.405	41,113	17,728	2551	2156	–	C:98.4 [S:83.4, D:15.0], F:0.5, M:1.1
RAGNAROK	ref	26,272	4212	0.191	31,954	16,333	2103	910	–	C:97.8 [S:89.6, D:8.2], F:0.6, M:1.6
BRAKER3	OrthoDB	31,584	8406	0.363	42,033	17,079	2715	2667	202	C:98.2 [S:82.6, D:15.6], F:0.4, M:1.4
RAGNAROK	UniProt	24,867	4154	0.201	37,369	17,817	3406	825	163	C:97.2 [S:89.4, D:7.8], F:0.8, M:2.0
***Prunus persica***										
Prunus_persica_v2.0.a1	–	26,873	5130	0.236	–	–	–	–	369	C:98.6 [S:96.8, D:1.8], F:0.9, M:0.4
BRAKER3	ref	30,425	11,531	0.610	42,250	14,606	3435	6295	–	C:98.4 [S:84.2, D:14.3], F:0.6, M:1.0
RAGNAROK	ref	25,049	3847	0.181	31,873	16,995	3345	1558	–	C:99.2 [S:93.2, D:6.0], F:0.3, M:0.5
BRAKER3	OrthoDB	35,141	12,258	0.536	45,019	14,029	3960	7085	375	C:98.8 [S:81.7, D:17.1], F:0.3, M:0.8
RAGNAROK	UniProt	23,315	4003	0.207	40,517	14,434	4776	1238	309	C:98.6 [S:93.1, D:5.5], F:0.6, M:0.7
***Malus × domestica***										
gene_models_20170612	–	46,558	7356	0.188	–	–	–	–	474	C:96.8 [S:55.9, D:40.9], F:1.7, M:1.5
BRAKER3	ref	43,203	13,891	0.474	62,552	18,213	9464	6235	–	C:97.7 [S:46.6, D:51.1], F:0.8, M:1.5
RAGNAROK	ref	43,824	7917	0.220	40,893	21,999	7418	5923	–	C:98.1 [S:52.4, D:45.7], F:0.7, M:1.1
BRAKER3	OrthoDB	54,974	18,360	0.501	68,331	16,875	11,314	12,551	514	C:90.7 [S:51.0, D:39.7], F:1.4, M:7.8
RAGNAROK	UniProt	40,841	8273	0.254	56,833	16,740	9683	5636	533	C:98.0 [S:53.0, D:45.0], F:0.8, M:1.2
***Liriodendron chinense***										
Lich.1_0	–	35,269	468	0.013	–	–	–	–	309	C:73.8 [S:67.9, D:6.0], F:19.5, M:6.6
BRAKER3	ref	42,083	19,604	0.872	111,948	6398	12,819	18,248	–	C:81.3 [S:61.9, D:19.3], F:6.8, M:11.9
RAGNAROK	ref	29,930	3869	0.148	77,269	10,957	11,948	5925	–	C:76.9 [S:67.6, D:9.4], F:15.1, M:8.0
RAGNAROK (PacBio)	ref	28,226	3433	0.138	77,947	11,443	12,528	5387	–	C:79.2 [S:71.1, D:8.0], F:10.9, M:10.0
BRAKER3	OrthoDB	36,888	12,751	0.528	99,381	5389	14,285	11,313	524	C:87.1 [S:65.3, D:21.8], F:4.9, M:8.0
RAGNAROK	UniProt	27,853	5353	0.238	102,036	4458	14,386	5680	539	C:70.3 [S:61.7, D:8.6], F:15.4, M:14.3
***Solanum tuberosum***										
Stuberosum_686_v6	–	32,917	5571	0.204	–	–	–	–	537	C:93.8 [S:55.2, D:38.6], F:4.6, M:1.5
BRAKER3	ref	29,054	7028	0.319	49,991	16,957	5162	1535	–	C:94.3 [S:82.9, D:11.4], F:2.7, M:3.0
RAGNAROK	ref	33,663	5641	0.201	33,463	19,627	2844	4411	–	C:96.8 [S:89.1, D:7.7], F:2.1, M:1.0
BRAKER3	OrthoDB	30,015	8287	0.381	58,468	13,574	6306	3769	300	C:97.9 [S:87.1, D:10.8], F:0.7, M:1.4
RAGNAROK	UniProt	31,983	6132	0.237	49,720	14,775	4615	4427	343	C:96.4 [S:88.6, D:7.7], F:1.9, M:1.7
***Triticum aestivum***										
ChineseSpring_725_v2.1	–	106,914	26,947	0.337	–	–	–	–	2394	C:97.5 [S:1.7, D:95.9], F:1.2, M:1.3
BRAKER3	ref	110,355	44,815	0.684	178,974	42,271	26,241	28,543	–	C:96.6 [S:6.0, D:90.7], F:1.9, M:1.4
RAGNAROK	ref	119,223	25,073	0.266	135,428	46,321	24,308	36,077	–	C:96.3 [S:5.8, D:90.5], F:1.3, M:2.4
BRAKER3	OrthoDB	123,231	54,078	0.782	180,436	41,431	27,276	42,338	1827	C:97.1 [S:5.5, D:91.6], F:1.9, M:1.0
RAGNAROK	UniProt	116,600	25,670	0.282	151,589	40,883	26,622	35,714	2140	C:96.8 [S:6.6, D:90.2], F:1.4, M:1.8
***Zea mays***										
Zm‐B73 NAM‐5.0	–	39,756	8866	0.287	–	–	–	–	213	C:95.3 [S:30.3, D:65.1], F:2.6, M:2.1
BRAKER3	ref	43,198	18,122	0.723	86,024	17,611	8818	11,832	–	C:92.3 [S:72.9, D:19.3], F:3.7, M:4.1
RAGNAROK	ref	37,786	5905	0.185	65,114	21,299	8436	6234	–	C:93.6 [S:75.2, D:18.4], F:3.2, M:3.2
BRAKER3	OrthoDB	46,324	22,044	0.908	92,222	15,943	11,264	17,616	114	C:94.0 [S:74.2, D:19.8], F:2.7, M:3.3
RAGNAROK	UniProt	35,238	6096	0.209	80,741	17,682	10,860	6105	133	C:91.6 [S:74.1, D:17.5], F:4.3, M:4.0

Abbreviation: BUSCO, benchmarking universal single‐copy orthologs.

**FIGURE 4:**
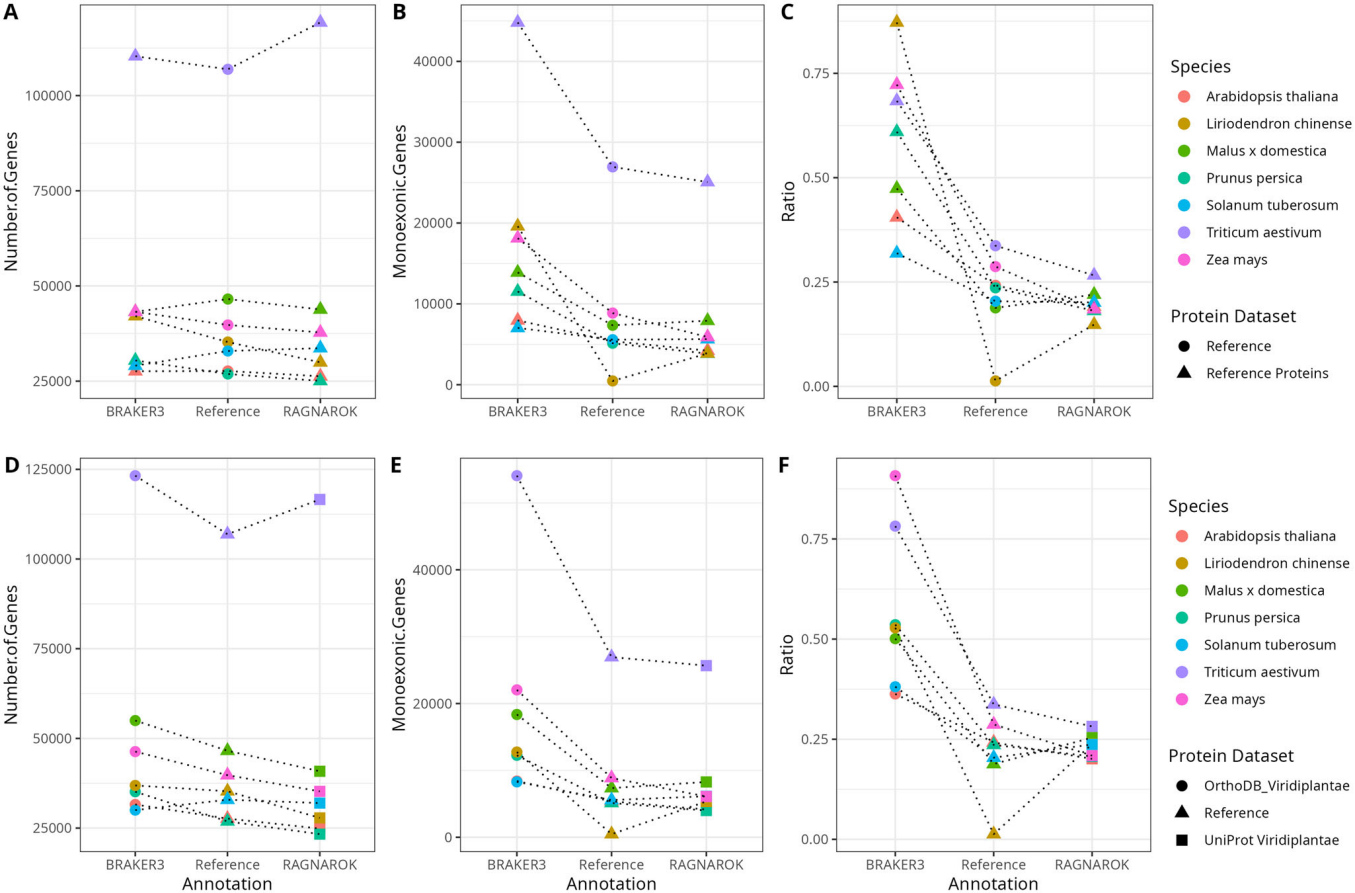
Annotation metrics between BRAKER3 and RAGNAROK compared to the reference annotation for seven genomes and two different protein dataset inputs. (A) Number of genes annotated using the reference protein database as input. (B) Number of mono‐exonic annotations using the reference protein database as input. (C) Ratio of mono:multi‐exonic annotations using the reference protein database as input. (D) Number of genes annotated using the Viridiplantae protein database as input. (E) Number of mono‐exonic annotations using the Viridiplantae protein database as input. (F) Ratio of mono:multi‐exonic annotations using the Viridiplantae protein database as input.

We also investigated the support for the novel loci in the BRAKER3 and RAGNAROK Viridiplantae protein database annotations. On average, BRAKER3 predicted more novel loci than RAGNAROK (Table 1; Wilcoxon *p‐*value = 0.0313). We sought to further investigate if there was support for these novel loci using two metrics: (1) if the predicted loci have high likelihood of genuine protein‐coding support using PSAURON (Sommer et al., 2025), and (2) if there was RNA‐seq coverage for the annotated features within the loci (Table S3). We found BRAKER3 and RAGNAROK were not significantly different in the percent of predicted peptides scored as not genuine at 12.7% and 9.8%, respectively. However, we did find RAGNAROK had a significantly lower percent of transcripts that lacked supportive RNA‐seq evidence at 42% compared to 52.9% for BRAKER3 (Wilcoxon *p‐*value = 0.0422) (Table S3). Taken together, these results suggest a marginal improvement in RAGNAROK when predicting novel transcripts, with the fewer novel transcripts predicted tending to have greater supporting evidence.

To further evaluate the performance of RAGNAROK compared to BRAKER3 beyond statistical metrics, we compared BUSCO (ODB12) values of the resulting transcriptomes from each annotation method to their reference genome's annotation (Table 1; Figure 5). BRAKER3 and RAGNAROK generally exhibited similar BUSCO completeness scores compared to the reference annotation for all seven species, regardless of the protein dataset used. Similar fragmented and missing BUSCOs were also observed between BRAKER3 and RAGNAROK. However, BRAKER3 was found to be significantly different in the number of missing genes compared to the reference annotation when using the reference protein dataset. However, this result is primarily influenced by the results of the *L. chinense* annotation (Table 1; Figure 5). We additionally used Resistify to identify the *R‐*genes annotated between each annotation method and the respective reference genome annotation (Table 1). Overall, neither pipeline was found to be significantly more effective at annotating *R‐*genes, even when comparing against the reference.

**FIGURE 5:**
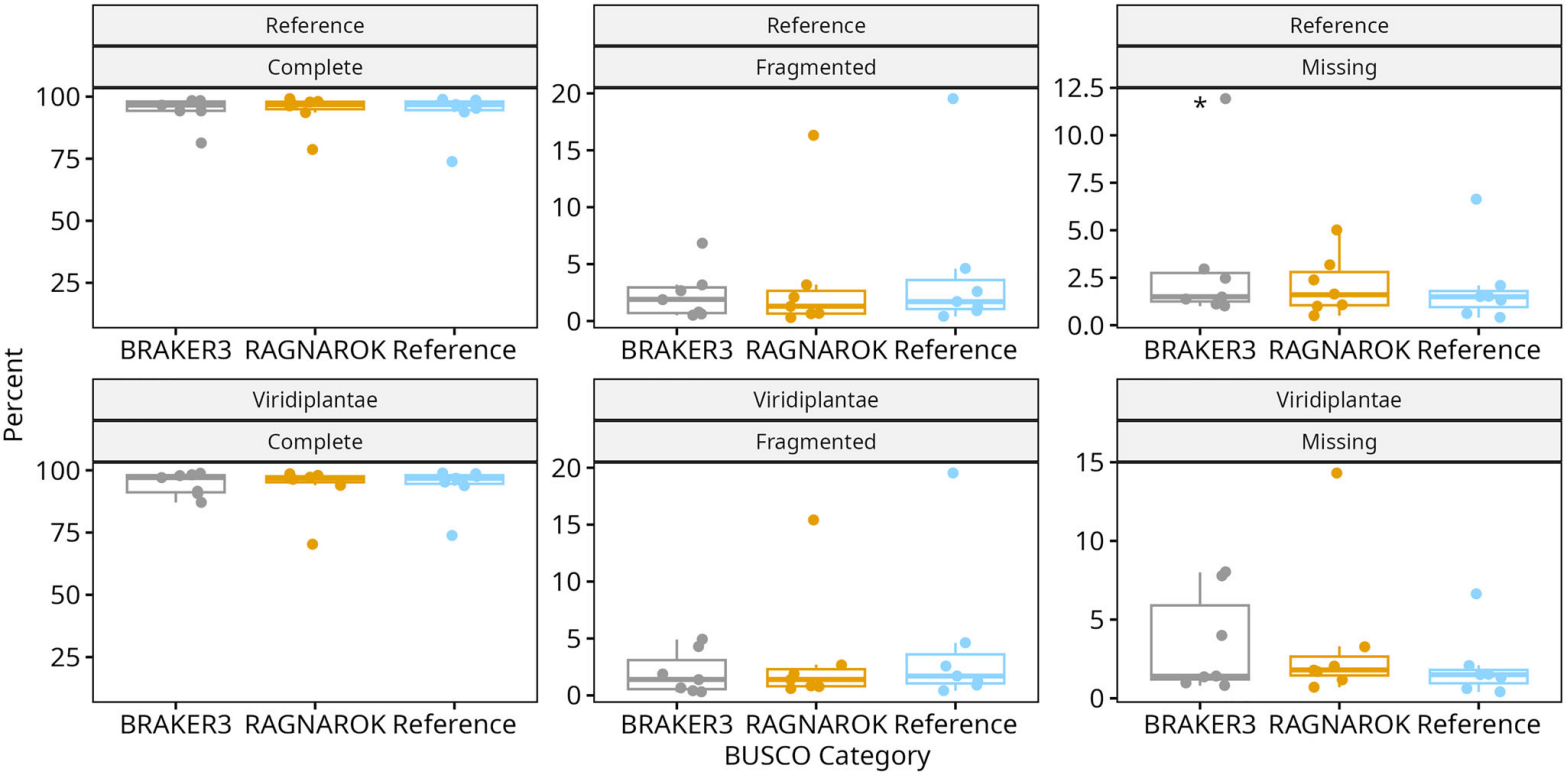
Comparisons of benchmarking universal single‐copy orthologs (BUSCO) scores of BRAKER3, RAGNAROK, and the reference annotations. Asterisks denote significant differences in means (Wilcoxon *p* ≤ 0.05).

### 3.2 Improvement in re‐annotation of MAKER‐annotated genomes

Our group and others have published and/or released several MAKER‐generated annotations for Rosaceae crops (Gottschalk et al., 2026; Mansfeld, Ou, et al., 2023; Mansfeld, Yocca, et al., 2023; Yocca et al., 2024). We took this opportunity to re‐annotate these five genome assemblies using RAGNAROK to compare against the original MAKER annotations (Table 2). RAGNAROK generated fewer gene annotations than MAKER in all five genomes. However, the difference was not significant (*p‐*value* = *0.0547). RAGNAROK also annotated significantly higher mono‐exonic genes compared to MAKER (*p‐*value = 0.00781). The resulting ratio of mono:multi‐exonic genes was significantly lower in the MAKER annotations compared to RAGNAROK (*p‐*value = 0.00647) (Table 2). When comparing BUSCO scores using the Rosaceae (ODB12) specific databases, RAGNAROK consistently outperformed MAKER (Figure 6). MAKER produced significantly lower BUSCO complete, as well as significantly higher fragmented and missing BUSCO genes (Figure 6).

**TABLE 2:** Re‐annotation statistics between MAKER and RAGNAROK.

Species	Genome version	Haplotype	Annotation	Number of genes	Mono‐exonic genes	Mono:multi‐exonic	BUSCO Rosaceae ODB12
*Malus angustifolia*	1.0	Hap1	MAKER	43,949	3255	0.080	C:89.6%[S:33.1%,D:56.5%],F:0.3%,M:10.1%,n:10071
*Malus angustifolia*	1.0	Hap2	MAKER	47,334	2677	0.060	C:89.8%[S:33.3%,D:56.5%],F:0.2%,M:10.0%,n:10071
*Malus angustifolia*	1.0	Hap1	RAGNAROK	43,329	10,443	0.318	C:96.3%[S:32.6%,D:63.7%],F:0.2%,M:3.6%,n:10071
*Malus angustifolia*	1.0	Hap2	RAGNAROK	42,239	10,384	0.326	C:96.2%[S:33.2%,D:63.0%],F:0.2%,M:3.6%,n:10071
*Malus fusca*	1.0	Hap1	MAKER	46,300	4452	0.106	C:93.0%[S:33.5%,D:59.5%],F:0.5%,M:6.5%,n:10071
*Malus fusca*	1.0	Hap2	MAKER	45,509	4572	0.112	C:92.9%[S:33.7%,D:59.1%],F:0.4%,M:6.7%,n:10071
*Malus fusca*	1.0	Hap1	RAGNAROK	40,202	6336	0.187	C:96.1%[S:31.1%,D:64.9%],F:0.2%,M:3.7%,n:10071
*Malus fusca*	1.0	Hap2	RAGNAROK	40,201	6339	0.187	C:96.2%[S:31.3%,D:64.9%],F:0.2%,M:3.6%,n:10071
*Prunus persica* ‘Lovell 2D*’*	3.0	–	MAKER	26,834	4081	0.179	C:95.3%[S:95.1%,D:0.2%],F:1.0%,M:3.6%,n:10071
*Prunus persica ‘*Lovell 2D*’*	3.0	–	RAGNAROK	28,372	5659	0.249	C:98.7%[S:93.8%,D:4.9%],F:0.1%,M:1.2%,n:10071
*Prunus persica ‘*Lovell 5D*’*	1.0	–	MAKER	26,764	3184	0.135	C:96.3%[S:92.2%,D:4.0%],F:0.9%,M:2.8%,n:10071
*Prunus persica ‘*Lovell 5D*’*	1.0	–	RAGNAROK	27,284	4443	0.195	C:98.6%[S:87.6%,D:11.0%],F:0.2%,M:1.3%,n:10071
*Pyrus communis*	2.3.a1	Hap1	MAKER	44,558	4110	0.102	C:94.0%[S:33.2%,D:60.8%],F:0.3%,M:5.7%,n:10071
*Pyrus communis*	2.3.a1	Hap2	MAKER	44,349	4143	0.103	C:94.1%[S:33.3%,D:60.7%],F:0.3%,M:5.6%,n:10071
*Pyrus communis*	1.0	Hap1	RAGNAROK	42,682	6056	0.165	C:94.9%[S:33.0%,D:61.9%],F:0.2%,M:4.9%,n:10071
*Pyrus communis*	1.0	Hap2	RAGNAROK	42,265	6160	0.171	C:95.1%[S:33.7%,D:61.4%],F:0.2%,M:4.7%,n:10071

Abbreviation: BUSCO, benchmarking universal single‐copy orthologs.

**FIGURE 6:**
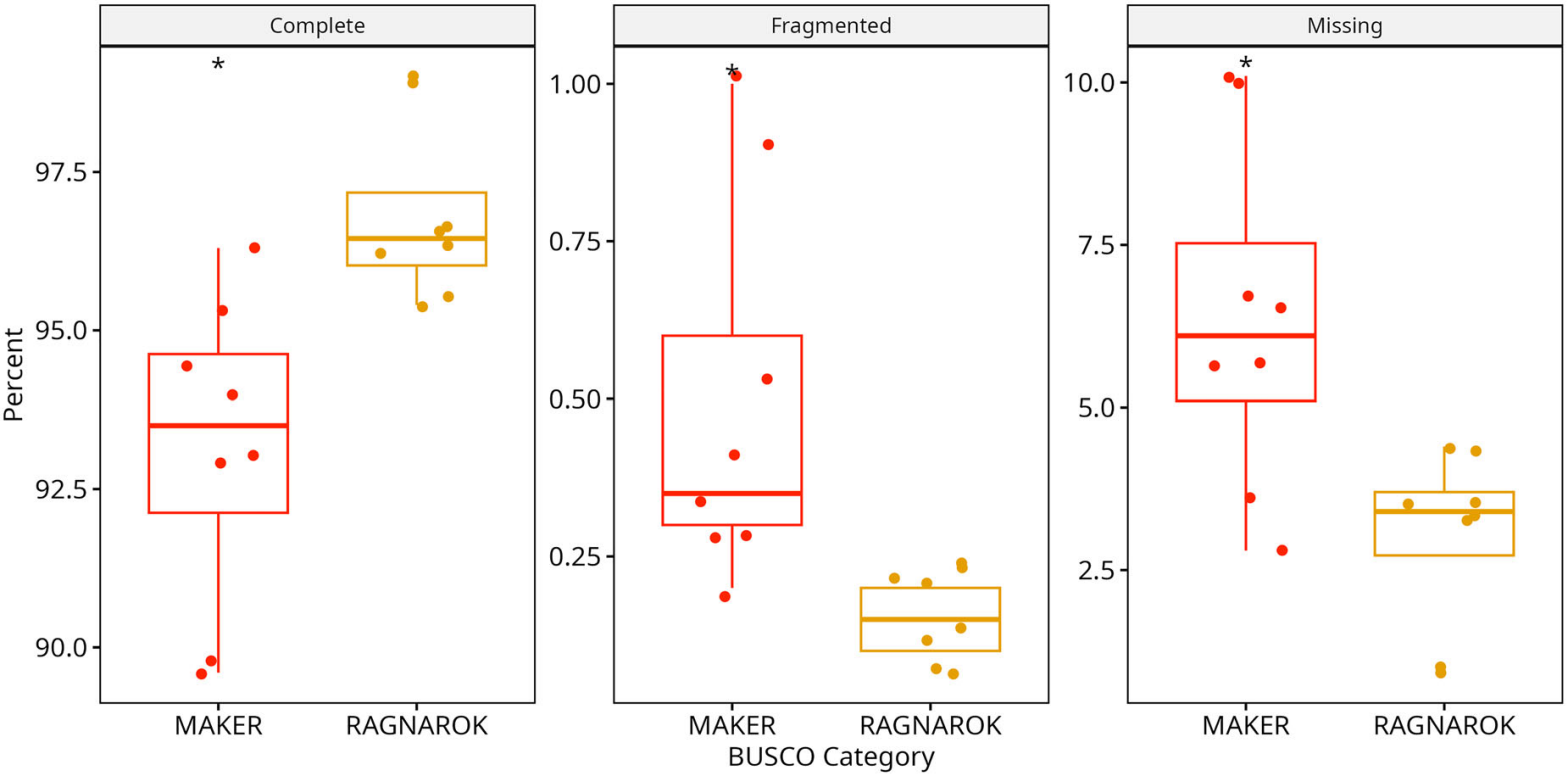
Comparisons of the improvements when re‐annotating genomes from MAKER to RAGNAROK. Asterisks denote significant differences in means using a Wilcoxon test, *p ≤ 0.05.

### 3.3 Run‐time comparisons

We recorded the wall and CPU times for annotating each dataset with BRAKER3 and RAGNAROK. For comparisons, we did not consider the time allocated to TE annotation via EDTA in RAGNAROK to provide a comparable comparison with BRAKER3, which does not include a TE annotation feature (Table 3). Helixer wall times are included in the wall time results, but reported separately for CPU hours, as this step runs in parallel on a dedicated GPU and cannot be accurately represented in CPU‐hour equivalents. Overall, RAGNAROK was faster than BRAKER3, regardless of genome size (Figure 7). Both BRAKER3 and RAGNAROK run times scaled linearly, but the slope of the regression model for RAGNAROK was not significantly lower than BRAKER3 (*p‐*value* = *0.0953). When comparing the CPU time models (Figure 7), we found that RAGNAROK was on average faster than BRAKER3 and that the interaction between annotation method and genome size was found to be significant (analysis of variance [ANOVA] *p‐*value = 7.24e‐07). Furthermore, the annotation method was a significant factor in influencing the CPU times (ANOVA *p‐*value = 4.07e‐04). The slope of the CPU time regression model for RAGNAROK was also significantly lower than BRAKER3 (*p‐*value < 0.0001).

**TABLE 3:** Run‐time comparisons between RAGNAROK and BRAKER3.

		Repeat annotation time (wall time)		Gene annotation time (wall time)		Gene annotation time (CPU time)		Helixer annotation time (wall time)	
Species	Annotation	H	Min	H	Min	H	Min	H	Min
*Arabidopsis thaliana*	BRAKER3			4	0	24	2		
*Arabidopsis thaliana*	RAGNAROK	5	1	4	22	66	0		37
*Prunus persica*	BRAKER3			4	32	19	41		
*Prunus persica*	RAGNAROK	9	44	3	37	64	46	1	17
*Malus* x *domestica*	BRAKER3			10	30	60	43		
*Malus* x *domestica*	RAGNAROK	26	40	7	46	172	41	3	29
*Liriodendron chinense*	BRAKER3	EDTA ran outside of RAGNAROK		24	34	58	23		
*Liriodendron chinense*	RAGNAROK			14	23	180	30	11	1
*Liriodendron chinense*	RAGNAROK (long‐reads)			15	14				
*Solanum tuberosum*	BRAKER3			5	16	177	41		
*Solanum tuberosum*	RAGNAROK			3	53	58	58	1	26
*Triticum aestivum*	BRAKER3			51	42	2160	56		
*Triticum aestivum*	RAGNAROK			35	10	405	38	22	58
*Zea mays*	BRAKER3			17	32	548	47		
*Zea mays*	RAGNAROK			8	31	108	1	4	41

*Note*: Gene annotation wall times include all steps in the RAGNAROK pipeline. Gene annotation central processing unit (CPU) times do not include Helixer, as it is graphics processing unit (GPU)‐based. Helixer wall times reported for comparison.

**FIGURE 7:**
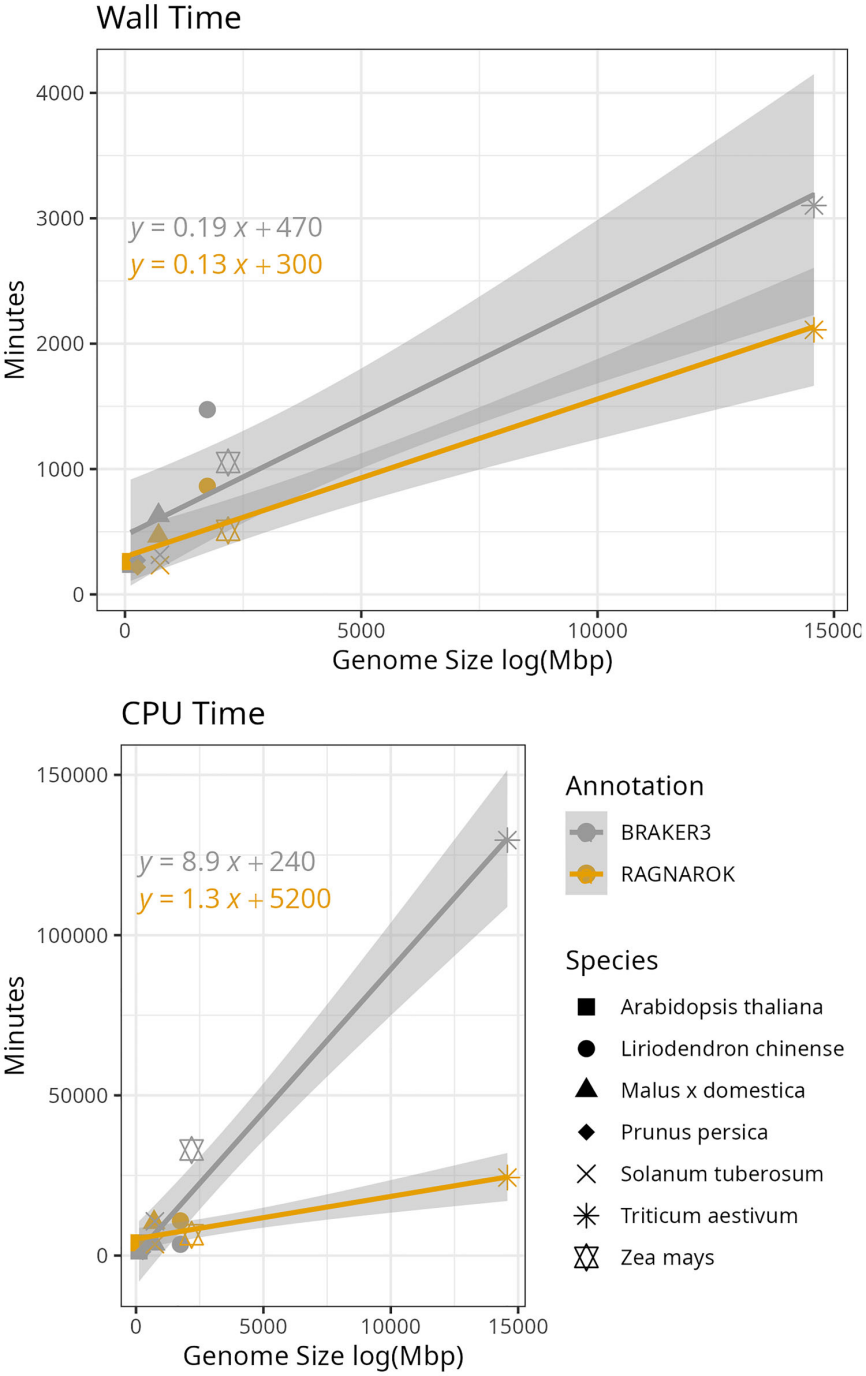
Comparing the run times between BRAKER3 and RAGNAROK annotation pipelines on seven datasets. Regression lines were determined using a linear model.

## 4 DISCUSSION

### 4.1 Installation

We aimed to develop an annotation pipeline that is easy to operate and install. Several other annotation pipelines, such as BRAKER3 and Funannotate, offer a user‐friendly installation method using Docker or Apptainer images (Gabriel, Brůna, et al., 2024; Kurtzer et al., 2017; Merkel, 2014; Palmer & Stajich, 2020). Historically, both of these pipelines require users to manually download and install the GeneMark predictor, which requires a license agreement and continually updated permission keys to operate (Brůna et al., 2024). However, a recent implementation of BRAKER3 using Docker has resolved this issue. MAKER requires local installation of the pipeline and all of its dependencies (Campbell et al., 2014). To overcome these hurdles and allow for greater accessibility, we elected to rely on Nextflow and Apptainer to manage operations and virtual installation of the dependencies for RAGNAROK (Kurtzer et al., 2017; Langer et al., 2025). To install and run RAGNAROK, a user will only be required to have Nextflow and Apptainer installed on their system. Furthermore, Nextflow facilitates ease of scalability, which allows RAGNAROK to be run on local machines or on a high‐performance computing cluster (HPCC) (Langer et al., 2025).

### 4.2 Performance against other annotation pipelines

To compare performance against BRAKER3, the current state of the art in genome annotation, we annotated seven reference‐quality genomes (Gabriel, Brůna, et al., 2024). These genomes include the model plant *A. thaliana* (119.6 Mb), two fruit crops, peach and apple (224.6 and 643.2 Mb, respectively), autotetraploid potato (741.6 Mb), and the gigabase‐scale genomes of maize, wheat, and *L. chinense* (Table 1) (J. Chen et al., 2019; Daccord et al., 2017; Hufford et al., 2021; Lamesch et al., 2012; Pham et al., 2020; Verde et al., 2013, 2017; Zhu et al., 2021). We undertook a similar approach as the BRAKER3 authors by limiting the amount of input data to reflect real‐world conditions (Gabriel, Brůna, et al., 2024). We found that RAGNAROK produced a higher level of exon, transcript, and gene‐level annotation accuracy compared to BRAKER3 using this strategy (Figure 2). In most annotation levels (exon, transcript, and gene), RAGNAROK produced significantly higher complete features (Table S2). As a result, RAGNAROK produced significantly higher transcript and gene F1 scores (Table S2). Furthermore, RAGNAROK demonstrated fewer mono‐exonic gene annotations than BRAKER3 (Table 2; Figure 4). Other authors have found similar high rates of mono‐exonic gene annotations when using BRAKER (Venturini et al., 2018). We observed mono:multi‐exonic ratios of ∼0.17 with RAGNAROK, which is close to the suggested ideal ratio of 0.2 for plants (Jain et al., 2008; Venturini et al., 2018). Several of the reference annotations used for benchmarking were previously generated using BRAKER software, which may have contributed to the observed concordance in gene model predictions in maize and the general trend of mono‐exonic:multi‐exonic ratios greater than 0.2 in the reference (Table 1). However, BRAKER was not the sole tool used in generating these annotations. In all cases except *A. thaliana* and *Z. mays*, RAGNAROK achieved higher in the complete and fragmented BUSCO scores over BRAKER3 (Table 1; Figure 5). This result could be attributed to methodological consistency of these annotations with those found in the BUSCO lineage databases, including mono‐exonic genes. This will require further analysis to conclude. Nevertheless, both BRAKER3 and RAGNAROK exhibited lower BUSCO complete scores compared to the reference annotation for *A. thaliana, P. persica, and Z. mays*. However, the differences were very small, with the maximum difference in complete gene percentage being <2% (Table 1). This is not a surprising result, as all three of these species have undergone manual curation to their annotation and utilized more extrinsic evidence during their annotation (Hufford et al., 2021; Lamesch et al., 2012; Verde et al., 2013, 2017). Because RAGNAROK and BRAKER3 handle protein evidence differently, each pipeline required a different protein database, precluding a perfectly symmetrical comparison. A small fraction of proteins in the databases used by either pipeline may derive from the reference organisms themselves. Table S4 reports the proportion of annotations supported by the different lines of evidence in RAGNAROK as well as the number of input protein sequences originating from the genus matching the reference genome for all annotations. Given the abundance of Arabidopsis‐based evidence in our UniProt database, we also report results for RAGNAROK annotation of *A. thaliana* with these proteins removed (Tables S2 and S4). Stability in the Mikado compare metrics suggests that helixer was able to recover loci previously predicted using miniprot‐based evidence. In contrast to previous BRAKER3 benchmarks, our comparisons against the model plant references reported lower sensitivity and precision. This reflects our inclusion of untranslated region (UTR) features, which constitute a substantial and functionally important part of a gene model that BRAKER3 does not predict. When UTRs were disregarded and only CDS models were compared, BRAKER3 showed higher gene‐level sensitivity, with values comparable to prior reports (Gabriel, Brůna, et al., 2024; Zimin et al., 2026). At the CDS level, RAGNAROK more frequently achieved higher precision, likely reflecting its more conservative output and smaller number of overall gene predictions (Table S5). We do not, however, regard UTRs as an artifact of scoring but as essential features for RNA‐seq quantification, the prediction and modulation of regulatory function, and the resolution of multiple isoforms at a shared locus.

We also compared RAGNAROK to MAKER annotations for a set of Rosaceae genomes (Gottschalk et al., 2026; Mansfeld, Ou, et al., 2023; Mansfeld, Yocca, et al., 2023; Yocca et al., 2024). Since these were not reference genome annotations, sensitivity, precision, and F1 scores could not be assessed. However, we did carry out comparisons on annotated gene number, mono‐exonic annotations, mono:multi‐exonic ratios, and BUSCO completeness. In contrast to the comparisons with BRAKER3, MAKER produced significantly fewer mono‐exonic gene annotations than RAGNAROK. However, the average ratio for MAKER (∼0.11) was below the suggested ratio of 0.2 (Jain et al., 2008; Venturini et al., 2018), whereas RAGNAROK maintained a ratio of ∼0.19. When comparing BUSCO scores between MAKER and RAGNAROK, RAGNAROK consistently outperformed MAKER (Figure 6; Table 2). This result was demonstrated using the >10k Rosaceae‐specific genes in the ODB12 BUSCO dataset (Tegenfeldt et al., 2025). Here, RAGNAROK achieved a completeness score averaging ∼96.8% compared to 93.2% for MAKER—a significant increase (Figure 6).

### 4.3 Run‐time

RAGNAROK run times were significantly faster overall than BRAKER3 across seven plant genomes of various lengths, as determined by linear regression (Figure 7). We observed slightly faster BRAKER3 runtimes than what was demonstrated in the BRAKER3 publication (Gabriel, Brůna, et al., 2024). For example, that publication reported a BRAKER3 runtime of 5 h 37 m for *A. thaliana* compared to the 4 h 0 m report in this study (Gabriel, Brůna, et al., 2024). This improvement could be associated with revisions to the BRAKER3 pipeline since its original release. However, the resource allocation limits between BRAKER3 and RAGNAROK may also favor RAGNAROK. RAGNAROK can be scaled to whatever is available on the local system or HPCC, and some of the subprocesses (i.e., Helixer and PSAURON) are GPU‐accelerated.

### 4.4 Scalability in the post‐genomics age

With the increased accessibility to producing high‐quality genome assemblies, extremely large projects, such as the Earth BioGenome Project, have arisen (Larivière et al., 2024). These projects aim to sequence and assemble hundreds of thousands to millions of genomes. The Earth BioGenome Project aims to assemble all ∼1.8 million eukaryotic species and has established a framework for this within the Galaxy ecosystem (The Galaxy Community, 2024), which would enable rapid, reproducible, and scalable solutions to genome assembly for this extremely large undertaking. However, existing tools fail to scale to meet the computational demands and requisite generalizability required to achieve such a project. Thus, the approach to annotating those genomes has yet to be developed (Larivière et al., 2024). More scalable and accelerated pipelines, such as RAGNAROK, could be a suitable option for those groups to deploy for the Viridiplantae if the required data inputs exist. RAGNAROK strikes an attractive balance between speed, sensitivity, precision, and limiting of potential over‐annotations. However, with further development, GPU‐accelerated gene annotation software, such as Helixer and Tiberius, could be suitable alternative options in data‐deficient organisms (Gabriel, Becker, et al., 2024; Holst et al., 2025).

### 4.5 Future directions

We aim to introduce further improvements and broaden the usage of RAGNAROK in future versions. Currently, we have limited RAGNAROK to plants but anticipate adding flexibility for other eukaryotes, such as animals and fungi. This will be achieved through the implementation of user‐selected Helixer models released by the software's developers for other taxonomic groups (Holst et al., 2025; Stiehler et al., 2021). Additionally, there are further opportunities to allow user‐generated Helixer models trained on organism‐specific RNA‐seq data. Similarly, we envision adding support for additional annotation software (e.g., Tiberius) (Gabriel, Becker, et al., 2024) to continue to leverage the advantages of both traditional and deep‐learning‐based approaches. The modular implementation of RAGNAROK and the parallel processing efficiency of Nextflow will continue to support the ongoing development and expansion of automated pipeline features. Nevertheless, the current implementation of RAGNAROK is already capable of incorporating gene models from such emerging tools with the addition of manual weighting from the end‐user.

We also plan to incorporate additional subparameters to enhance user‐specified intron length during the RNA‐seq alignment via STAR (Dobin et al., 2013). Currently, intron size is hard‐coded at a 10 kb max intron size to support what is commonly observed in plants. However, additional flexibility will be required for use in other eukaryotes. For example, when annotating a fungal genome, the max intron length should be reduced from the current 10 kb to ∼2 kb (Kupfer et al., 2004). We also plan to integrate the FindPlantNLR pipeline as an additional annotation process (S. H. Chen et al., 2023), which is currently available as an experimental feature. We have found that FindPlantNLR offers improvements in annotating complex nucleotide‐binding domain LRR proteins (NLRs), which are commonly found in complex tandem duplications (Gottschalk et al., 2026; Larson et al., 2025; Liu et al., 2025).

We also envision additional run time improvements. For example, there are other emerging GPU‐accelerated implementations of standard bioinformatic pipelines through initiatives such as Parabricks from NVIDIA (Santa Clara, CA) (Franke & Crowgey, 2020). Tasks such as RNA‐seq mapping using STAR and minimap have already been made available through Parabricks and would be complementary to the GPU‐accelerated Helixer gene prediction already implemented in RAGNAROK.

## AUTHOR CONTRIBUTIONS

**Ryan D. Kuster**: Formal analysis; methodology; software; writing—review and editing. **Zane C. Smith**: Methodology; validation; writing—review and editing. **Lauren Whitt**: Methodology; validation; writing—review and editing. **Margaret Staton**: Methodology; validation; writing—review and editing. **Ben N. Mansfeld**: Methodology; validation; writing—review and editing. **Christopher Gottschalk**: Conceptualization; formal analysis; methodology; validation; writing—original draft; writing—review and editing.

## CONFLICT OF INTEREST STATEMENT

The authors declare no conflicts of interest.

## Supporting information



Supplemental Table 1. Genomes, reference annotations, evidence datasets, and versions for different species used in the comparisons between annotation pipelines.

Supplemental Table 2. Precision and sensitivity for gene predictions by RAGNAROK and BRAKER3 compared to the reference annotation for seven reference quality genomes.

Supplemental Table 3. Novel annotation evidence statistics between RAGANAROK and BRAKER3 annotations using the Viridiplantae databases as protein input on seven reference quality genomes.

Supplemental Table 4. Evidence line breakdown for RAGNAROK annotations for seven reference quality genomes (st = StringTie, hx = Helixer, mp = miniprot, tr = TransDecoder).

Supplemental File 1. Commands executed in the comparisons between RAGNAROK, BRAKER3, and MAKER.

Supplementary Material

## Data Availability

To download and utilize the RAGNAROK pipeline, please refer to its repository on GitHub at the following web address: https://github.com/ryandkuster/ragnarok.
