# Supplementary material for: Ragnarok: A flexible and RApid GeNe annotation (ROcKs) pipeline deployed through Nextflow
Source: Plant Genome. 2026 Nov 14;19(4):e70338. doi: 10.1002/tpg2.70338 (PMC13769401; doi:10.1002/tpg2.70338)
Supplement: Supplementary file 1 [file TPG2-19-e70338-s002.pdf]

```
        --threads 96 \
        --workingdir=$RESULTS/braker3_db \
        --AUGUSTUS_CONFIG_PATH=$DERIVED/config \
        --genome=$masked_genome \
        --species=new \
        --prot_seq=$protein \
        --rnaseq_sets_ids=$ill_list \
        --rnaseq_sets_dirs=$ill \
        &> braker.log
```

Convert gtf to gff3

```
braker_gtf=$RESULTS/braker3_db/braker.gtf

gtf2gff.pl \
    <${braker_gtf} \
    --gff3 \
    --out=${RESULTS}/braker3_db/braker.gff3
```

Run Mikado stats

```
out_dir=$RESULTS/annotation_metrics
mkdir -p $out_dir

braker_gff=$RESULTS/braker3_db/braker.gff3
ragnarok_gff=$RESULTS/ragnarok_db/publish/RAGNAROK/ragnarok_db.entap_filte
red.gff3

singularity exec -B $PROJ docker://gemygk/mikado:v2.3.5rc2 \
    mikado util stats $braker_gff $out_dir/braker3_stats.txt

singularity exec -B $PROJ docker://gemygk/mikado:v2.3.5rc2 \
    mikado util stats $ragnarok_gff $out_dir/ragnarok_stats.txt

singularity exec -B $PROJ docker://gemygk/mikado:v2.3.5rc2 \
    mikado util stats $ref_gff $out_dir/ref_stats.txt
```

Run Mikado compare

```
out_dir=$RESULTS/annotation_metrics
mkdir -p $out_dir

braker_gff=$RESULTS/braker3_db/braker.gff3
ragnarok_gff=$RESULTS/ragnarok_db/publish/RAGNAROK/ragnarok_db.entap_filte
red.gff3

singularity exec -B $PROJ docker://gemygk/mikado:v2.3.5rc2 \
    mikado compare \
        -r $ref_gff \
        -p $ragnarok_gff \
        -o $out_dir/compare_ragnarok_ref_stats.txt \
        -x 30

singularity exec -B $PROJ docker://gemygk/mikado:v2.3.5rc2 \
    mikado compare \
        -r $ref_gff \
        -p $braker_gff \
        -o $out_dir/compare_braker3_ref_stats.txt \
        -x 30
```

Run BUSCO metrics

```
out_dir=$RESULTS/annotation_metrics_busco
mkdir -p $out_dir

braker_gff=$RESULTS/braker3_db/braker.gff3
ragnarok_gff=$RESULTS/ragnarok_db/publish/RAGNAROK/ragnarok_db.entap_filte
red.gff3

# Retrieve the transcript sequences from the final gff files.
singularity exec -B $PROJ docker://quay.io/biocontainers/gffread:0.12.7--
h077b44d_6 \
    gffread \
        -w $out_dir/braker3.transcripts.fa \
        -g $genome \
        $braker_gff

singularity exec -B $PROJ docker://quay.io/biocontainers/gffread:0.12.7--
h077b44d_6 \
    gffread \
        -w $out_dir/ragnarok.transcripts.fa \
        -g $genome \
        $ragnarok_gff

singularity exec -B $PROJ docker://quay.io/biocontainers/gffread:0.12.7--
h077b44d_6 \
    gffread \
        -w $out_dir/ref.transcripts.fa \
        -g $genome \
        $ref_gff

# Run BUSCO with ODB12
singularity exec -B $PROJ docker://quay.io/biocontainers/busco:5.8.2--
pyhdfd78af_0 \
    busco \
        -m transcriptome \
        -i $out_dir/braker3.transcripts.fa \
        -c 36 \
        --out_path $out_dir/busco_braker3_v12 \
        -l embryophyta_odb12

singularity exec -B $PROJ docker://quay.io/biocontainers/busco:5.8.2--
pyhdfd78af_0 \
    busco \
        -m transcriptome \
        -i $out_dir/ragnarok.transcripts.fa \
        -c 36 \
        --out_path $out_dir/busco_ragnarok_v12 \
        -l embryophyta_odb12

singularity exec -B $PROJ docker://quay.io/biocontainers/busco:5.8.2--
pyhdfd78af_0 \
    busco \
        -m transcriptome \
        -i $out_dir/ref.transcripts.fa \
        -c 36 \
        --out_path $out_dir/busco_ref_v12 \
        -l embryophyta_odb12
```

Run PSAURON

```
out_dir=$RESULTS/annotation_metrics_psauron
mkdir -p $out_dir

braker_gff=$RESULTS/braker3_db/braker.gff3
ragnarok_gff=$RESULTS/ragnarok_db/publish/RAGNAROK/ragnarok_db.entap_filte
red.gff3

# Retrieve the protein sequences from the final gff files.
singularity exec -B $PROJ docker://quay.io/biocontainers/gffread:0.12.7--
h077b44d_6 \
    gffread \
        -y $out_dir/braker3.proteins.fa \
        -g $genome \
        $braker_gff

singularity exec -B $PROJ docker://quay.io/biocontainers/gffread:0.12.7--
h077b44d_6 \
    gffread \
        -y $out_dir/ragnarok.proteins.fa \
        -g $genome \
        $ragnarok_gff

singularity exec -B $PROJ docker://quay.io/biocontainers/gffread:0.12.7--
h077b44d_6 \
    gffread \
        -y $out_dir/ref.proteins.fa \
        -g $genome \
        $ref_gff

# Run PSAURON.
singularity exec -B $PROJ docker://phuongdoan96/psauron:1.0.8 \
    psauron \
        -p \
        -i $out_dir/braker3.proteins.fa \
        -o $out_dir/braker_psauron.txt

singularity exec -B $PROJ docker://phuongdoan96/psauron:1.0.8 \
    psauron \
        -p \
        -i $out_dir/ragnarok.proteins.fa \
        -o $out_dir/ragnarok_psauron.txt

singularity exec -B $PROJ docker://phuongdoan96/psauron:1.0.8 \
    psauron \
        -p \
        -i $out_dir/ref.proteins.fa \
        -o $out_dir/ref_psauron.txt
```

Analyze novel genes

```
out_dir=$RESULTS/annotation_metrics_novel_genes
mkdir -p $out_dir

bam=$RESULTS/ragnarok_db/publish/alignments/sorted_bam/short_sorted_merged
.bam

ragnarok_gff=$RESULTS/ragnarok_db/publish/RAGNAROK/ragnarok_db.entap_filte
red.gff3
ragnarok_tmap=$RESULTS/annotation_metrics/compare_ragnarok_ref_stats.txt.t
map
ragnarok_psauron=$RESULTS/annotation_metrics_psauron/ragnarok_psauron.txt

braker_gff=$RESULTS/braker3_db/braker.gff3
braker_tmap=$RESULTS/annotation_metrics/compare_braker3_ref_stats.txt.tmap
braker_psauron=$RESULTS/annotation_metrics_psauron/braker_psauron.txt

# Ragnarok novel genes
apptainer exec -B $PROJ docker://quay.io/biocontainers/stringtie:3.0.0--
h29c0135_0 \
    stringtie \
        -p 32 \
        -e \
        -G $ragnarok_gff \
        -o $out_dir/rag_novel_exp.gtf \
        $bam

# Get all rows where n_f1 is 0
tail -n +2 $ragnarok_tmap | \
    awk -F'\t' '$10+0 == 0' | \
    cut -f 5 | \
    sort | \
    uniq > $out_dir/rag_novel_genes.txt

tail -n +2 $ragnarok_tmap | \
    awk -F'\t' '$10+0 == 0' | \
    cut -f 4 | \
    sort | \
    uniq > $out_dir/rag_novel_tx.txt

python3 novel_gene_filter.py \
    -g $out_dir/rag_novel_exp.gtf \
    -l $out_dir/rag_novel_genes.txt \
    -o $out_dir/rag_novel_exp_filter.gtf


# Braker3 novel genes
apptainer exec -B $PROJ docker://quay.io/biocontainers/stringtie:3.0.0--
h29c0135_0 \
    stringtie \
        -p 32 \
        -e \
        -G $braker_gff \
        -o $out_dir/braker_novel_exp.gtf \
        $bam

# Get all rows where n_f1 is 0
tail -n +2 $braker_tmap | \
    awk -F'\t' '$10+0 == 0' | \
    cut -f 5 | \
    sort | \
    uniq > $out_dir/braker_novel_genes.txt

tail -n +2 $braker_tmap | \
    awk -F'\t' '$10+0 == 0' | \
    cut -f 4 | \
    sort | \
    uniq > $out_dir/braker_novel_tx.txt

python3 novel_gene_filter.py \
    -g $out_dir/braker_novel_exp.gtf \
    -l $out_dir/braker_novel_genes.txt \
    -o $out_dir/braker_novel_exp_filter.gtf

grep -Ff $out_dir/rag_novel_tx.txt $ragnarok_psauron | grep -c False
tail -n +3 $out_dir/rag_novel_exp_filter.gtf | awk '$14 ~ /^"0\.0+";$/' |
wc -l

grep -Ff $out_dir/braker_novel_tx.txt $braker_psauron | grep -c False
tail -n +3 $out_dir/braker_novel_exp_filter.gtf | awk '$14 ~ /^"0\.0+";$/'
| wc -l
```

Analyze R-genes using Resistify

```
ref_prots=$RESULTS/annotation_metrics_psauron/ref.proteins.fa
braker_prots=$RESULTS/annotation_metrics_psauron/braker3.proteins.fa
ragnarok_prots=$RESULTS/annotation_metrics_psauron/ragnarok.proteins.fa

out_dir=$RESULTS/annotation_metrics_resistify
mkdir -p $out_dir
mkdir -p $out_dir/ref
mkdir -p $out_dir/braker
mkdir -p $out_dir/ragnarok

eval "$(conda shell.bash hook)"
# Resistify version 2.1.0
conda activate resistify

resistify nlr \
    --threads 20 \
    $ref_prots \
    -o $out_dir/ref

resistify nlr \
    --threads 20 \
    $braker_prots \
    -o $out_dir/braker

resistify nlr \
    --threads 20 \
    $ragnarok_prots \
    -o $out_dir/ragnarok
```

Narrow annotations to CDS-only

```
source config

mkdir -p $DERIVED/gtf_conversions

while IFS=, read -r name gff gtf ref_gff ; do
    echo $name

    # BRAKER

##########################################################################
######
    # convert braker3 gtf to cds only gff
    awk 'BEGIN{FS=OFS="\t"} $3=="CDS"{print; $3="exon"; print}' $gtf | \
        apptainer exec -B $PROJ
docker://quay.io/biocontainers/gffread:0.12.9--hf426362_0 \
            gffread - --keep-genes -o
$DERIVED/gtf_conversions/braker_${name}_cds.gff

    # REF

##########################################################################
######
    # convert ref gff to gtf
    apptainer exec -B $PROJ docker://quay.io/biocontainers/gffread:0.12.9-
-hf426362_0 \
        gffread \
            -T \
            $ref_gff \
            -o $DERIVED/gtf_conversions/ref_${name}.gtf

    # convert ref gtf to cds only gff
    awk 'BEGIN{FS=OFS="\t"} $3=="CDS"{print; $3="exon"; print}'
$DERIVED/gtf_conversions/ref_${name}.gtf | \
        apptainer exec -B $PROJ
docker://quay.io/biocontainers/gffread:0.12.9--hf426362_0 \
            gffread - --keep-genes -o
$DERIVED/gtf_conversions/ref_${name}_cds.gff

    # RAGNAROK

##########################################################################
######
    # convert rag gff to gtf
    apptainer exec -B $PROJ docker://quay.io/biocontainers/gffread:0.12.9-
-hf426362_0 \
        gffread \
            -T \
            $gff \
            -o $DERIVED/gtf_conversions/rag_${name}.gtf

    # convert rag gtf to cds only gff
    awk 'BEGIN{FS=OFS="\t"} $3=="CDS"{print; $3="exon"; print}'
$DERIVED/gtf_conversions/rag_${name}.gtf | \
        apptainer exec -B $PROJ
docker://quay.io/biocontainers/gffread:0.12.9--hf426362_0 \
            gffread - --keep-genes -o
$DERIVED/gtf_conversions/rag_${name}_cds.gff

done < revision_files.txt
```

Quantify proteins in databases

```
source config

braker_db=$DATA/db/ortho_db/Viridiplantae.fa
odb_species=$DATA/db/ortho_db/odb12v2_species.tab
ragnarok_db=$DATA/db/uniprotkb_taxonomy_id_33090_AND_reviewe_2026_02_02.fa
sta

mkdir -p $DERIVED/ortho_db
mkdir -p $DERIVED/uniprot

# Get the counts of each ncbi taxid in the orthodb viridiplantae fasta.
#grep "^>" $braker_db | cut -f 2 | sort | uniq -c >
$DERIVED/ortho_db/Viridiplantae_species.txt

echo -e "genus\thits" > $DERIVED/uniprot_hits_summary.txt
echo -e "genus\thits" > $DERIVED/ortho_db_hits_summary.txt

for genus in arabidopsis prunus malus liriodendron triticum solanum zea ;
do
    echo $genus
    hits=$( grep "^>" $ragnarok_db | grep -P -i "OS=${genus}\b" | wc -l )
    echo -e "${genus}\t${hits}" >> $DERIVED/uniprot_hits_summary.txt
    grep "^>" $ragnarok_db | grep -P -i "OS=${genus}\b" >
$DERIVED/uniprot/${genus}_hits.txt

    grep -P -i "\t${genus}\b" $odb_species | cut -f 2 >
$DERIVED/ortho_db/${genus}_ids.txt
    hits=$( grep -w -F -f $DERIVED/ortho_db/${genus}_ids.txt $braker_db |
wc -l )
    echo -e "${genus}\t${hits}" >> $DERIVED/ortho_db_hits_summary.txt
done
```

Quantify mRNA features by evidence line:

```
import sys

from collections import Counter

gff = sys.argv[1]

result = []

with open(gff) as f:
    for line in f:
        if line.startswith("#"):
            continue
        fields = line.rstrip().split("\t")
        if not fields[2] == "mRNA":
            continue
        info = fields[8]
        for entry in info.split(";"):
            if entry.startswith("alias"):
                result.append(entry.split("=")[1][:2])

result.sort()

counts = Counter(result)
total = len(result)
print(f"Total length: {total}")
for string, count in counts.items():
    print(f"  {string}: {count} ({count/total:.1%})")
```

Short read accessions

Arabidopsis thaliana

```
SRR8714016
SRR8759751
SRR4010853
SRR7289569
SRR12547664
SRR12076896
```

Prunus persica

```
SRR531865
SRR531863
SRR531864
SRR531862
```
